# Diversity and Co-Occurrence Patterns of Fungal and Bacterial Communities from Alkaline Sediments and Water of Julong High-Altitude Hot Springs at Tianchi Volcano, Northeast China

**DOI:** 10.3390/biology10090894

**Published:** 2021-09-10

**Authors:** Xiao Wang, Lorenzo Pecoraro

**Affiliations:** School of Pharmaceutical Science and Technology, Tianjin University, 92 Weijin Road, Tianjin 300072, China; wang_xiao1996@163.com

**Keywords:** fungi, bacteria, microbial community, hot springs, extreme environments, microorganism diversity, morphology, Illumina sequencing, microbial network analysis

## Abstract

**Simple Summary:**

Volcanic hot springs are considered of particular interest in biology as precious sources of peculiar microorganisms adapted to extreme environmental conditions. The Julong high-altitude volcanic hot springs in northeast China are characterized by alkali, sulfur and metal enrichment. The microbial communities colonizing these intriguing habitats still remain completely unknown. We carried out the present study in order to shed light on the Julong hot springs fungal and bacterial community diversity, structure and interactions, and to understand the impact of pH on the microorganisms colonizing the investigated environment. We detected a large variety of typical photosynthetic, thermophilic, alkalophilic, antimicrobial-active, and sulfide- and metal-oxidizing microbial taxa representing clear links to the extreme properties of the studied environment. Results showed a striking microorganism community variation strongly influenced by pH under clearly alkaline conditions. Our analyses suggests that mutualistic relationships might be common among microorganisms coexisting in the Julong hot springs, especially for the bacterial community. This study provides new insights in the diversity and ecological interactions of microorganisms living in high-altitude volcanic hot springs and contributes to our knowledge of abiotic factors influencing the microbial community in the analyzed extreme environment.

**Abstract:**

The Julong high-altitude volcanic hot springs in northeast China are of undeniable interest for microbiological studies due to their unique, extreme environmental conditions. The objective of this study was to provide a comprehensive analysis of the unexplored fungal and bacterial community composition, structure and networks in sediments and water from the Julong hot springs using a combination of culture-based methods and metabarcoding. A total of 65 fungal and 21 bacterial strains were isolated. Fungal genera *Trichoderma* and *Cladosporium* were dominant in sediments, while the most abundant fungi in hot spring water were *Aspergillus* and *Alternaria*. Bacterial communities in sediments and water were dominated by the genera *Chryseobacterium* and *Pseudomonas*, respectively. Metabarcoding analysis revealed significant differences in the microorganism communities from the two hot springs. Results suggested a strong influence of pH on the analyzed microbial diversity, at least when the environmental conditions became clearly alkaline. Our analyses indicated that mutualistic interactions may play an essential role in shaping stable microbial networks in the studied hot springs. The much more complicated bacterial than fungal networks described in our study may suggest that the more flexible trophic strategies of bacteria are beneficial for their survival and fitness under extreme conditions.

## 1. Introduction

Microorganisms are essential components in all ecosystems. Given their small size, easy dispersal, flexibility to utilize a broad range of nutrients, and ability to tolerate extreme environmental conditions, microorganisms are capable of colonizing habitats that are unfavorable for the majority of life forms [1]. Microorganisms that thrive under extreme conditions (e.g., hot, cold, alkaline, and acidic environments) are generally referred to as extremophiles and specifically grouped as thermophiles, psychrophiles, alkaliphiles, and acidophiles based on their particular environmental preference [2,3]. The rare and unique physicochemical and geographical characteristics of extreme environments allow extremophiles to evolve peculiar physiological properties as an adaptation mechanism [4]. As a consequence, studies on microbial communities inhabiting extreme environments are likely to reveal the presence of novel, exclusive species that can be exploited as sources of bioactive compounds for pharmaceutical and industrial applications [5,6,7]. Moreover, extremophilic microbes are of particular interest as they provide suitable models for the study of the origin and evolution of life [4].

Geothermally heated hot springs are examples of extreme environments that harbor interesting and peculiar extremophiles [8,9]. In addition to the unusual high temperature, various dissolved minerals, such as magnesium, calcium, sodium, chlorides, sulfates, or silica, generally distinguish geothermal water from nongeothermal groundwater [10], thus supporting the presence of distinct microbial communities. For decades, researchers have been exploring the microbial diversity of thermal ecosystems in different continents worldwide, including the near-boiling silica-depositing thermal springs at Yellowstone National Park in the USA [11], hot springs with a wide range temperature (22–75 °C) across the Tibetan Plateau of China [12], intertidal hot springs of Iceland [13], acidic thermal pools of Russia [14], and alkaline hot springs of Kenya [15], using traditional culture-dependent methods [16,17,18], as well as recently developed high-throughput sequencing [15,18]. Novel thermophilic, alkaliphilic, and metal-tolerant species able to produce valuable biotechnological products, such as antibiotics [8], bioethanol [19], and thermostable enzymes [20], have been frequently discovered from hot springs [5,19,21].

An increasing number of studies have attempted to establish the link between microbial community structure/diversity and physicochemical conditions, such as temperature, pH, and water chemistry [22,23,24], in order to better understand microbial ecology and functions in geothermal areas. Among different environmental parameters, the effect of pH has received particular attention. Previous studies suggested that pH is one of the fundamental physicochemical factors affecting microbial diversity in hot spring environments [23,25]. For instance, Li et al. [26] found a correlation between increasing pH and higher archaeal phylogenetic diversity in alkaline hot spring systems in southwest China, although the abundance of archaea was lower at high pH compared to acidic conditions. Huo and coauthors [27] showed that pH was a crucial parameter driving the differences in bacterial and archaeal communities between two analyzed hydrothermal areas of the Tengchong hot spring system in Yunnan Province, China. However, the majority of recent studies investigating geothermal environments mainly focused on the diversity of bacterial and archaeal communities [12,13,14,16,17,18], while the diversity and function of fungal communities received much more limited attention [15,25,28].

The Julong volcanic hot springs are sulfur-enriched hot springs [29,30] distributed across the north flank of the active Tianchi volcano in Changbai Mountain Nature Reserve, northeast China, at an altitude of about 1900 m a.s.l. Julong geothermal spring water is enriched with minerals such as Na, Ca, Mg, Fe, Cu, and Mn [30]. Trace components such as H_2_SiO_3_, Li, and Sr are also abundant [30,31]. The geothermal fluid in the Julong hot springs is characterized by temperatures between 70 and 83 °C [32] and pH from weakly alkaline to alkaline (7.44–8.17) [33]. Gases related to mantle magmatic activity, which are released from the hot spring area, include CO_2_, N_2_, H_2_, He, and CH_4_, with CO_2_ being the most abundant [31,34]. Sinter caked in the hot spring region is expected to host unusual thermophilic biota that may provide clues to ancient life on Earth and possibly Mars [35]. Although the Julong geothermal springs are of undeniable interest for microbiological studies, due to their unique, extreme environmental characteristics, the microbial communities colonizing these habitats still remain completely unknown. A few studies recording novel bacterial species have been performed on the hot springs in Changbai Mountain [29,36]. For instance, an extremely thermophilic, cellulolytic, and hydrogen-producing novel bacterium, *Caldicellulosiruptor changbaiensis*, was isolated from hot spring sediments collected at an altitude of 2189 m a.s.l. [36]. Cai et al. discovered a novel thermophilic anaerobic bacterium, *Fervidobacterium changbaicum*, from a mixture sample of water and mud collected in a hot spring from an undescribed location within the Changbai Mountains [29].

The objective of this study was to provide a comprehensive analysis of the fungal and bacterial community composition, structure, and networks in alkaline sediments and water from the unexplored high-altitude Julong volcanic hot springs. The microbiological exploration of these habitats, which were expected to harbor peculiar microorganisms adapted to the particularly extreme environmental conditions, was performed using a combination of molecular and morphological identification of culturable microbes and metabarcoding based on Illumina sequencing. Our aim was also to evaluate the impact of pH on the microbial diversity colonizing the studied environment. The results of this study fill in some of the blanks of our knowledge of bacterial and fungal community ecology in the Julong hot springs and expand our current understanding of microbial networks in hot spring habitats.

## 2. Materials and Methods

### 2.1. Description of the Sample Sites and Measurement of pH Values

**Figure 1:**
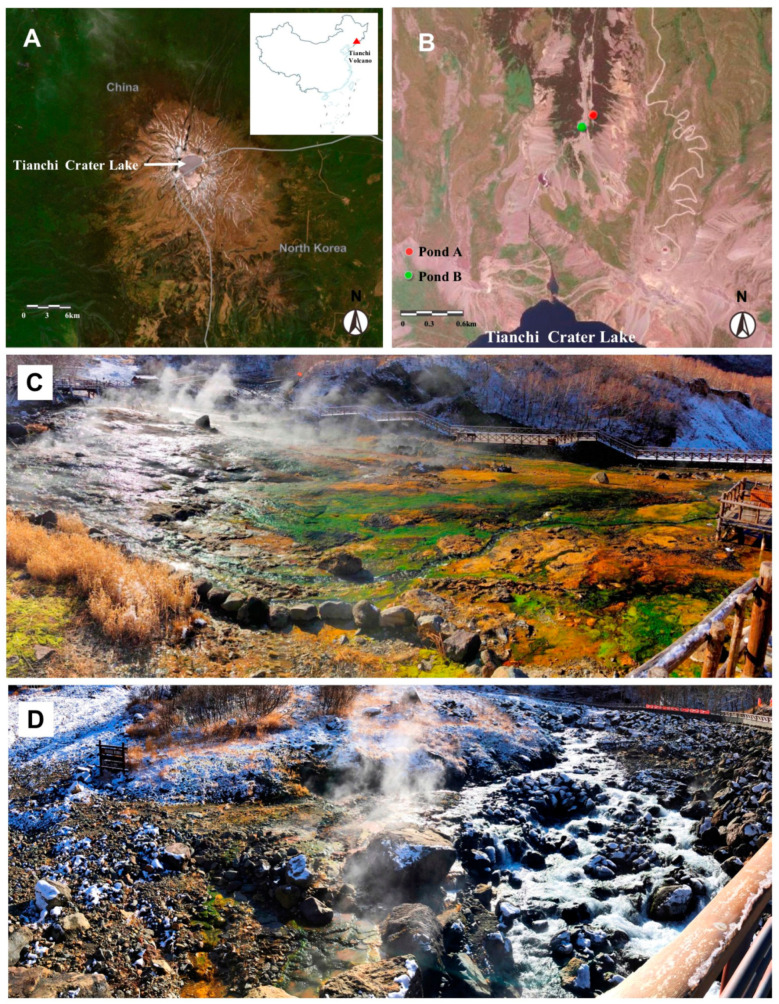
(**A**) Location of Tianchi Volcano in China and vertical view; (**B**) detailed location of the sampling area on the northern side of Tianchi Volcanic cone; (**C**) the main pond system: Pond A; (**D**) the smaller spring area: Pond B. The maps were generated from ArcGIS Online (https://maps.arcgis.com/index.html, accessed on 31 March 2021).

Tianchi Volcano is located at the highest point of the Changbai Mountain range, straddling the border between China and North Korea (Figure 1A) [37]. The studied Julong hot springs are distributed in the northern valleys around the cone of the Tianchi Volcano. On 26 October 2019, sediment and water samples were collected from two pond systems of the Julong hot springs based on the pond scales (Figure 1B). Seven water and 11 sediment samples were collected randomly from the main pond system (pond A, 128°3′32″ E, 42°2′28″ N, 1890 m a.s.l.) (Figure 1C), while three water and nine sediment samples were collected from a smaller spring area separated from the main one by a river (pond B, 128°3′27″ E, 42°2′24″ N, 1880 m a.s.l.) (Figure 1D), resulting in a total of 10 water and 20 sediment samples for the hot spring complex. The samples were placed into sterilized Falcon 50 mL centrifuge tubes and immediately stored on ice in insulated containers. We used sterile rubber gloves to prevent cross-contamination. After returning to the laboratory, the 20 sediment samples were divided into two subsamples, one stored at 4 °C for isolation of culturable microbes and measurement of pH values, the other one stored at –80 °C for metabarcoding analysis. The 10 water samples were entirely stored at 4 °C for microbe isolation and pH measurement. The pH values of water and ground sediments diluted in water (1:2.5 *w*/*v*) from the Julong hot springs were measured by a pH meter (STARTER3100, OHAUS, NJ, USA) after shaking uniformly. 

### 2.2. Cultivable Microbe Isolation, Microscopy, and Molecular Analyses

The hot spring sediment samples were prepared following the method described in detail by Wang and Pecoraro [38]. In brief, five- and 50-times diluted suspensions were prepared from ground sediment materials and finally shaken at 220 rpm for 5 min. Afterward, 100 μL of uniform sediment suspension from each of the two dilutions and 100 μL of each water sample were plated on potato dextrose agar (PDA) amended with 100 mg/L penicillin and streptomycin to reduce the growth of bacteria to a minimum level and favor the isolation of fungi. Additionally, in order to access more possible microbes in the hot spring water, 100 μL of water from each sample was also plated on PDA without antibiotics to obtain bacterial strains. Petri dishes were sealed and incubated at room temperature (25 °C) in darkness. After microbial colonies appeared, the different morphotypes were accurately selected for strain isolation based on characteristics such as texture and pigmentation. Single colonies were picked and transferred to new Petri dishes containing the same medium to obtain pure cultures.

Fungal morphological traits (such as septate hyphae, phialides, hyphal coils, conidiophores, conidia, and conidial chains) were examined under a Nikon ECLIPSE Ci microscope for identification of isolates following the taxonomic keys for different taxa described by Gilman [39], Alexopoulos, and Mims [40].

Molecular identification of fungal and bacterial strains was performed based on PCR amplification of the internal transcribed spacer (ITS) region of fungal rDNA using primers ITS1 and ITS4, and V3 to V4 hypervariable region of bacterial 16S rRNA using primers U341F and U806R, respectively. Fungal and bacterial genomic DNA was obtained and amplified as described by Wang and Pecoraro [38]. PCR products were Sanger sequenced at Tianjin Tsingke Biological Technology Co., Ltd. (Tianjin, China). Sequences were assembled using BioEdit 7.0.9.0 [41]. All obtained sequences were BLASTn searched in NCBI and assigned to potential genera and species. The nomenclature followed Index Fungorum (indexfungorum.org, accessed on 10 March 2021). 

### 2.3. Culture-Independent Microbial Diversity and Statistical Analysis

Total genomic DNA from the sediment samples was extracted using a FastDNA^®^ Spin Kit for soil (MP Biomedicals, Solon, OH, USA) according to the manufacturer’s instructions. PCR amplifications were carried out using specific primers (fungal ITS2 rDNA region: ITS3F and ITS4R; V3–V4 hypervariable region of the bacterial 16S rRNA gene: 338F and 806R) as described by Wang and Pecoraro [38]. The amplicon libraries were constructed using the NEXTFLEX^®^ Rapid DNA-Seq Kit (Bioo Scientific, Austin, TX, USA) and paired-end sequenced (2 × 300 bases) on an Illumina MiSeq PE300 platform (Illumina Inc., San Diego, CA, USA) at Shanghai Majorbio Bio-Pharm Technology Co., Ltd. (Shanghai, China). Raw sequences were quality-filtered by Fastp v0.19.6 [42] according to the following rules: (1) filter the bases in the tail of the reads with a quality score below 20; (2) set 50 bp sliding windows on the reads, cut off the back-end bases from the window if the average quality value in the window is lower than 20, and the reads below 50 bp after quality control were removed; (3) reads containing ambiguous nucleotide (N) were eliminated; (4) the barcode mismatches were not allowed and the maximum number of primer mismatches was two. Paired-end reads were subsequently merged by FLASH v1.2.11 [43]. Chimeras were removed using UCHIME [44]. Operational taxonomic unit (OTU) clustering was performed using UPARSE v7.1 [45]. Taxonomic assigning was performed based on RDP Classifier v2.2 [46] against the UNITE Database v8.0 (for fungal sequences) and SILVA 16S rRNA Database (release 132, for bacterial sequences). Fungal and bacterial OTU tables were rarefied to the minimum sequencing depth counts up to 53,941 and 29,376 reads per sample for downstream analyses, respectively. Alpha diversity was calculated based on OTU numbers and the Shannon index in Mothur (version 1.30.1), and rarefaction curves were drawn in the R package. A between-groups Venn diagram was plotted using R to identify unique and common OTUs. A Wilcoxon rank-sum test was used to explore variations in Shannon diversity and OTU richness between different groups. The beta diversity of fungal and bacterial communities was calculated using Weighted Unifrac distances [47]. Principal coordinate analysis and analysis of similarity with 999 permutations were performed for the visualization and assessment of dissimilarities between samples based on a phylogenetic weighted Unifrac distance matrice. Linear regression was used to evaluate the relationships between pH and microbial richness.

### 2.4. Co-Occurrence Network Construction and Analysis

The interactions between microbial taxa were determined through a network structure to decipher the complexity of fungal and bacterial communities in the Julong hot springs. Co-occurrence networks of fungal and bacterial communities in the analyzed hot springs were constructed based on OTU relative abundance and inferred by calculating the Spearman correlation matrix between OTUs. For all networks, we retained the OTUs in the top 50 abundance level for analyses. The pairs of OTUs with a Spearman’s rank correlation value ≥ 0.7 or ≤ –0.7 and a significant *p* value (< 0.001) were identified and included for further network construction. The topological properties estimation (including the number of network nodes, edges, positive and negative correlations, average degree, network diameter, and average path length) and visualization of the network were conducted using Gephi (version 0.9.2) [48]. In the network diagrams, each node represents an OTU indicating an individual taxon, whereas the edges between every two nodes correspond to significant positive or negative correlations between those two taxa.

## 3. Results

### 3.1. pH Measurement of Water and Sediment Samples

**Figure 2:**
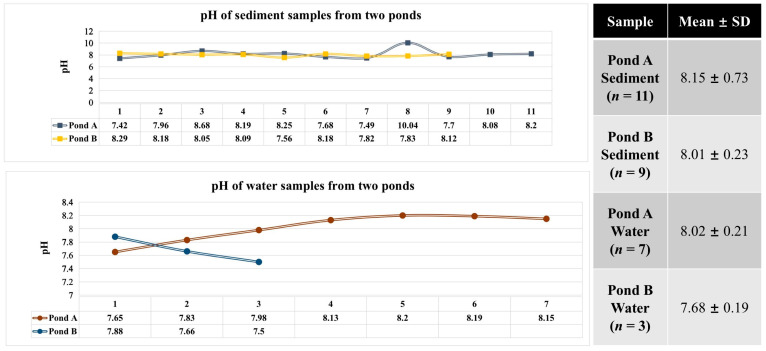
pH values of water and sediment from Pond A and Pond B of the Julong hot springs.

The pH was alkaline for all samples. For sediment samples, pH ranged from 7.42 to 10.04, while for water samples, from 7.50 to 8.20 (Figure 2). According to the mean ± SD values, for both sediments and water, Pond A was more alkaline than Pond B (Figure 2).

### 3.2. Diversity of Culturable Microbes

Based on combined molecular sequencing and microscopic identification from twenty sediment samples of the Julong hot springs, 51 fungal strains belonging to 31 species in 17 genera from Ascomycota (i.e., 30 species, 50 strains) and Mucoromycota (1 species, 1 strain) were isolated. *Trichoderma* (19.61%), *Cladosporium* (19.61%)*, Plectosphaerella* (9.80%)*, Acremonium* (7.84%), and *Aspergillus* (7.84%) were the most abundant genera (Table 1). Seven bacterial strains were isolated and identified as members of the genera *Chryseobacterium* (2 species, 3 strains), belonging to the phylum Bacteroidetes (42.86%), *Herbaspirillum* (2 species, 2 strains), *Sphingomonas* (1 strain), and *Pseudomonas* (1 strain), belonging to the phylum Proteobacteria (57.14%) (Table 1). From the 10 collected water samples, a total of 14 fungal strains (9 species in 6 genera) of Ascomycota (78.57%) and Basidiomycota (21.43%) were isolated. The most dominant genera were *Aspergillus* (42.86%) and *Alternaria* (21.43%) (Table 2). Water samples also yielded 14 bacterial isolates, which were identified in the genera *Bacillus* (1 strain) belonging to the phylum Firmicutes (7.14%), *Acinetobacter* (1), *Enterobacter* (1), and *Pseudomonas* (8 species, 11 strains), belonging to Proteobacteria (92.86%). *Pseudomonas* dominated the water bacterial community, accounting for 72.73% of the species diversity (Table 2). Details about sequence similarity, number, isolation source, and taxonomic identity of isolated strains are reported in Supplementary Tables S1–S5.

### 3.3. Microbial Diversity by Illumina Sequencing

From the analyzed 20 sediment samples, a total of 1,430,057 and 860,784 effective sequences were obtained by fungal ITS2 gene and bacterial 16S V3–V4 region sequencing, respectively. The number of fungal sequences from each sample ranged from 53,941 to 138,713, while for bacteria it ranged from 29,376 to 54,145. After normalization of sequences, 1533 OTUs could be identified as members of the fungal kingdom, into 11 phyla, 38 classes, 104 orders, 253 families, and 457 genera, while 4158 bacterial OTUs were assigned to 59 phyla, 126 classes, 331 orders, 554 families, and 962 genera.

The rarefaction curves related to the detected fungal and bacterial OTUs showed that most samples reached saturation (Figure S1A,C), and the total OTU accumulation curves of different ponds nearly approached an asymptote (Figure S1B,D), strongly supporting the idea that our data are representative of the whole fungal and bacterial diversity in the two analyzed pond systems of the Julong hot springs.

**Figure 3:**
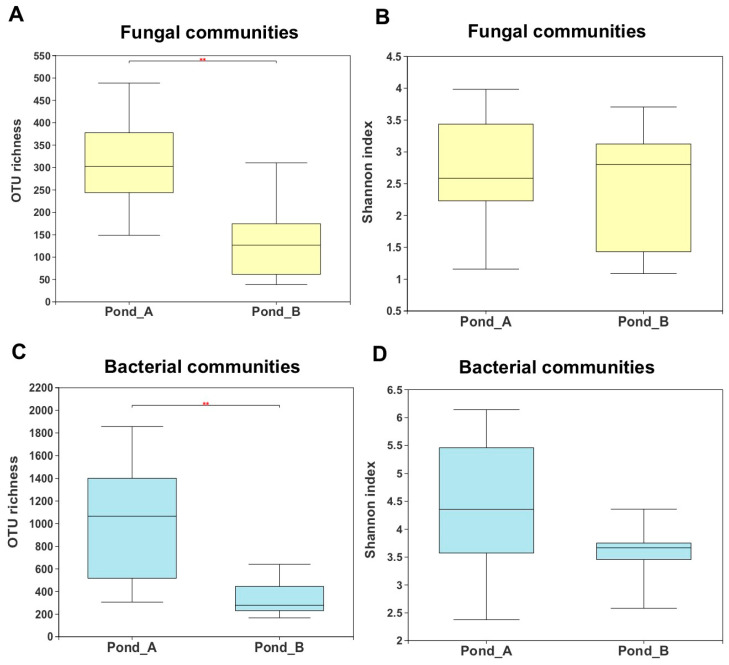
Sediment fungal and bacterial alpha diversity (OTU richness and Shannon index) in two ponds of the Julong hot springs. Difference between Pond A and Pond B as evaluated by a Wilcoxon rank-sum test is indicated as: ** *p* < 0.01. (**A**) Fungal OTU richness; (**B**) Fungal Shannon index; (**C**) Bacterial OTU richness; (**D**) Bacterial Shannon index.

From total samples, α-diversity indexes of bacterial communities were significantly higher than those of fungal communities (Table S6). Comparative analyses of α-diversity between the two hot spring ponds showed that Pond A harbored significantly higher numbers of fungal and bacterial OTUs (*p* < 0.01) than Pond B (Figure 3A,C). The lowest fungal OTU richness (38 OTUs) was detected in Pond B (sample B-S7), and the highest richness (488 OTUs) was detected from sample A-S6 from Pond A (Table S6). For the bacterial community, the lowest and highest OTU richness values were detected from Pond B (sample B-S8) and Pond A (sample A-S2), represented by 165 and 1854 OTUs, respectively (Table S6). For the Shannon index, however, no significant differences were detected between the two ponds (Figure 3B,D).

**Figure 4:**
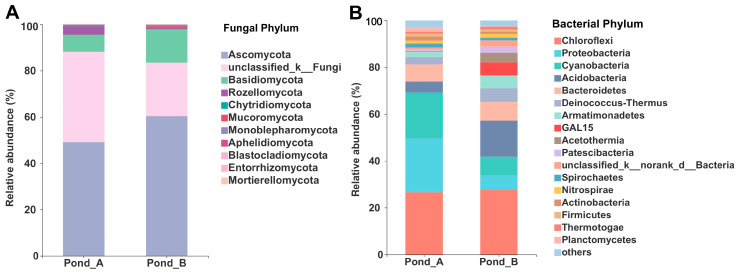
Phylum-level relative abundance of fungi (**A**) and bacteria (**B**) from the two analyzed ponds of the Julong hot springs. The bacterial phyla with very low relative abundances (<1%) were merged as “others” in the bar plot. In the *taxa* names, “d” = domain and “k” = kingdom.

Overall, the fungal phylum Ascomycota was dominant in the two ponds. Basidiomycota and Rozellomycota also represented a significant portion of the fungal community together with fungi unidentified at phylum level represented by 533 OTUs. Another seven fungal phyla were detected with smaller proportions (Figure 4A). For the bacterial community, the phyla Chloroflexi, Proteobacteria, Cyanobacteria, Acidobacteria, Bacteroidetes, Deinococcus–Thermus, Armatimonadetes, GAL15, Acetothermia, Patescibacteria, Spirochaetes, Nitrospirae, Actinibacteria, and Firmicutes were dominant in the studied hot springs together with a group of unidentified bacteria (Figure 4B). 

**Figure 5:**
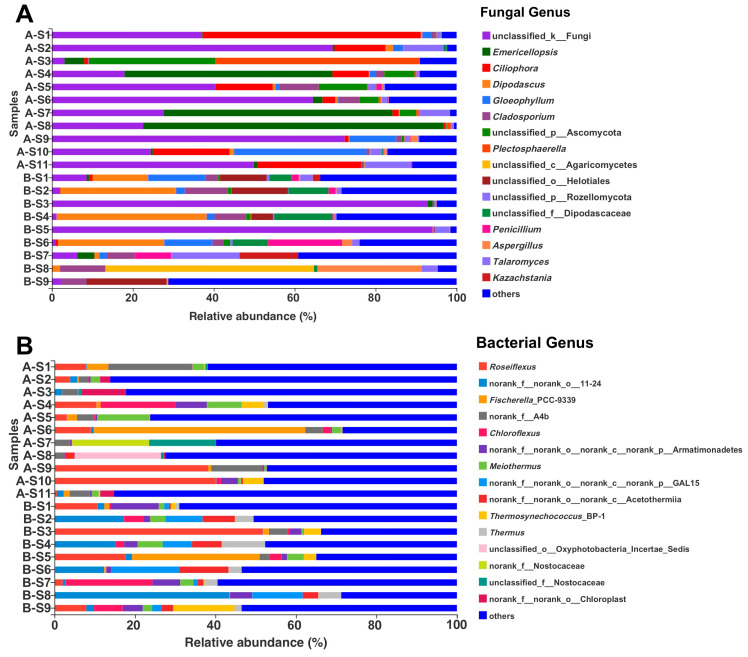
Genus-level relative abundance of fungi (**A**) and bacteria (**B**) from all samples. The relative abundance of fungal and bacterial genera <10% were merged as “others” in the bar plots. Samples A-S1–A-S11 were from Pond A and B-S1–B-S9 from Pond B. In the taxa names, “d” = domain, “k” = kingdom, “p” = phylum, “c” = class, “o” = order, and “f” = family.

At the genus level, *Emericellopsis*, *Ciliophora*, *Dipodascus*, *Gloeophyllum*, *Cladosporium*, *Plectosphaerella*, *Penicillium*, *Aspergillus*, *Talaromyces*, and *Kazachstania* dominated the studied hot spring fungal community. However, the most dominant fungal taxon remained not clearly identified (Figure 5A). Other taxa, only identified at either phylum (Ascomycota, Rozellomycota), class (Agaricomycetes), order (Helotiales), or family level (Dipodascaceae), were also abundant (Figure 5A). Regardless of unclassified groups, different fungal genera dominated the two analyzed ponds, being *Emericellopsis* and *Ciliophora* the most abundant in Pond A, and *Dipodascus* in Pond B (Figure 5A). The 16S rRNA analyses showed that the bacterial genera *Roseiflexus*, *Fischerella*, *Chloroflexus*, *Meiothermus*, *Thermosynechococcus*, and *Thermus* were dominant in the studied hot springs (Figure 5B). However, a large number (23.11%) of abundant bacterial reads remained unidentified.

**Figure 6:**
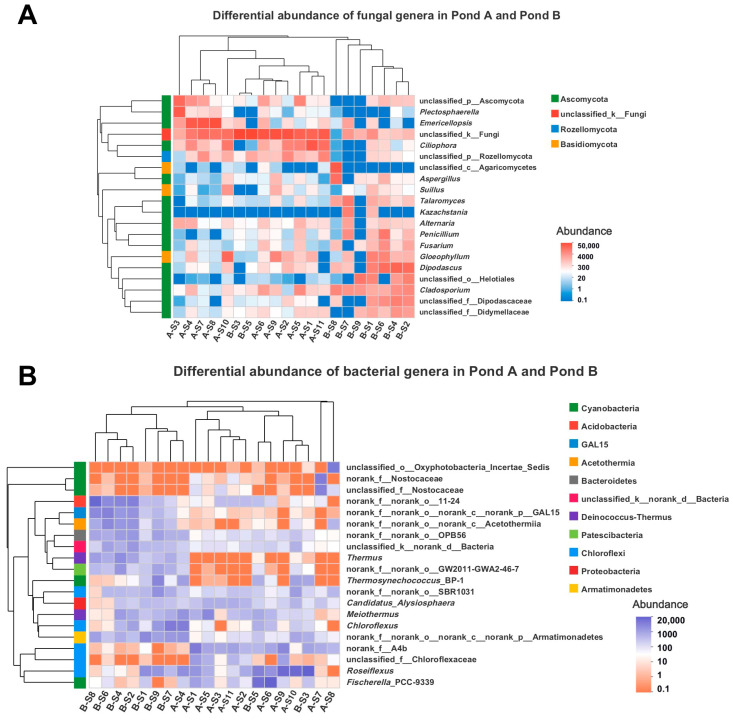
Heatmap of the top 20 abundant fungal (**A**) and bacterial (**B**) genera. Block color represents the abundance of different microbial genera. In the *taxa* names, “d” = domain, “k” = kingdom, “p” = phylum, “c” = class, “o” = order, and “f” = family.

**Figure 7:**
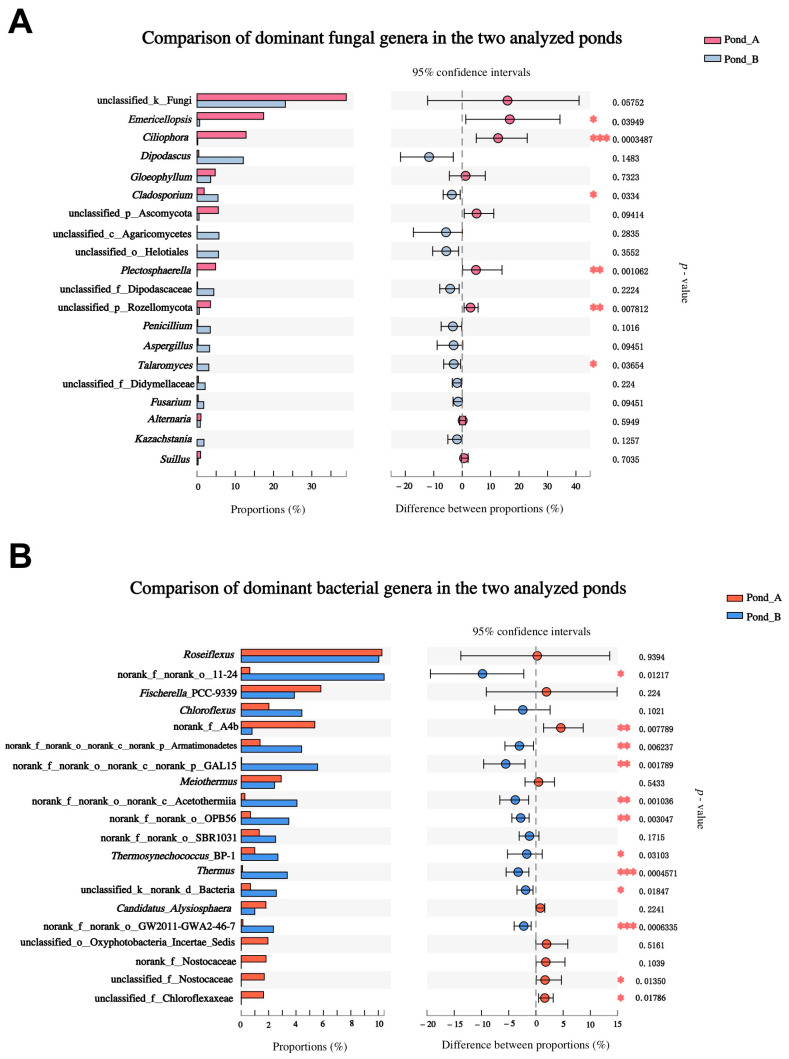
Comparison of the dominant genera of fungal (**A**) and bacterial (**B**) communities in the two analyzed ponds. Differences in the genera between both ponds as evaluated by Wilcoxon rank-sum test is indicated as: * *p* < 0.05, ** *p* < 0.01, *** *p* < 0.001. In the *taxa* names, “d” = domain, “k” = kingdom, “p” = phylum, “c” = class, “o” = order, and “f” = family.

Heatmaps and comparisons of the top 20 dominant microbial genera were performed to explore the composition similarity and the distinctive microbial genera in the two analyzed ponds (Figure 6 and Figure 7). Practically, for both fungal and bacterial communities, the samples from each pond could be clustered in the same branch (Figure 6). The abundances of the fungal genera *Emericellopsis*, *Ciliophora*, *Plectosphaerella*, and the unclassified taxon belonging to the phylum Rozellomycota were significantly higher in Pond A, while *Cladosporium* and *Talaromyces* were much more abundant in Pond B (Figure 6A and Figure 7A). The bacterial community showed a clearly distinguishable heatmap pattern (Figure 6B), as significant differences are shown in most of the dominant bacterial genera between the two ponds (Figure 7B). The bacterial genera *Thermosynechococcus* and *Thermus* presented significantly higher abundance in Pond B than Pond A (Figure 6B).

**Figure 8:**
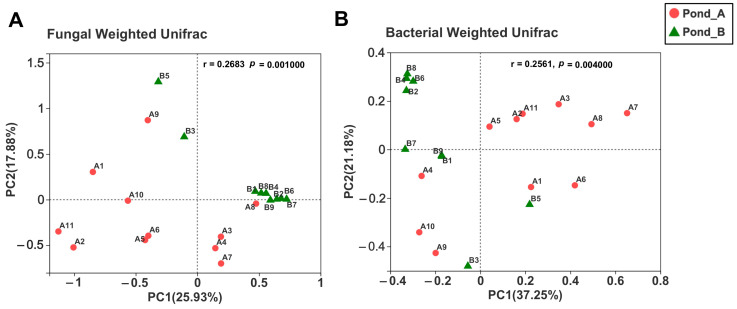
Principal coordinate analysis plots of fungal (**A**) and bacterial (**B**) communities in two hot spring ponds based on weighted Unifrac distance. The r and *p*-values of the analysis of similarity were shown respectively in each figure (*p* < 0.01).

Principal coordinate analysis revealed clustering of fungal and bacterial OTUs based on sampling locations (Pond A and Pond B) using weighted Unifrac distance (Figure 8), which was consistent with the heatmap results of sample clustering. 

**Figure 9:**
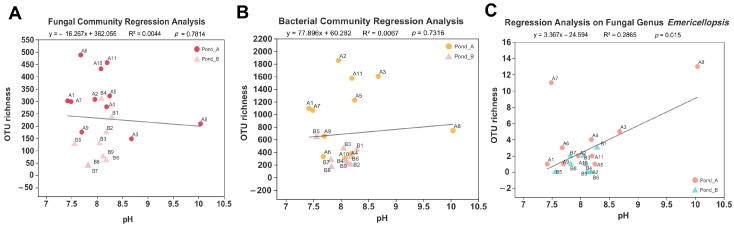
Linear regression between pH value and fungal OTU richness (**A**), bacterial OTU richness (**B**), and richness of OTU belonging to the fungal genus *Emericellopsis* (**C**).

Regression curves showed the relationships between sediment pH and OTU richness (Figure 9). No significant correlation existed between pH and overall fungal (R^2^ = 0.0044, *p* = 0.7814, Figure 9A) and bacterial OTU richness (R^2^ = 0.0067, *p* = 0.7316, Figure 9B). However, we noticed a significant correlation between pH and the richness of *Emericellopsis* fungi (R^2^ = 0.2865, *p* = 0.015, Figure 9C), which represented the most dominant fungal group. For this genus, the richness showed upward tendency with the increase of pH (Figure 9C), whereas for the general fungal community the richness decreased with the increase of pH (Figure 9A).

### 3.4. Fungal and Bacterial Co-Occurrence Networks

**Figure 10:**
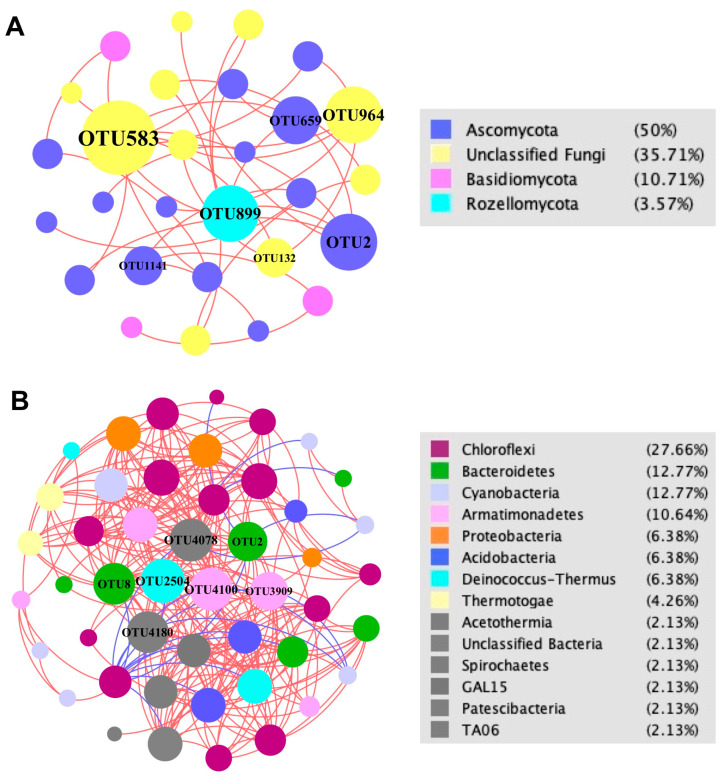
Sediment OTUs network analysis of the Julong hot springs (Fruchterman–Reingold layout). (**A**) Network of fungal community; (**B**) network of bacterial community. Each node represents an OTU indicating a single species. Color codes for nodes belonging to different dominant phyla. The node size is proportional to the degree (degree: number of direct correlations to a node). Positive interactions are displayed as red edges and negative interactions are displayed as blue edges.

Network analysis (Figure 10) showed that significant positive interactions dominated the microbial community of the Julong hot springs with an extremely high percentage of positive correlations in both fungal (100%) and bacterial (93%) communities (Table S7). The fungal network exhibited a very simple structure as reflected by a very low number of nodes and edges, as well as average degree (Figure 10A and Table S7). On the contrary, a much more complicated network structure was found for the bacterial community, with significantly different topological properties compared to the fungal network (Figure 10B and Table S7). Phyla Ascomycota, Basidiomycota, and Rozellomycota had the most abundant interactions with other nodes in the fungal network (Figure 10A). Bacterial phyla Patescibacteria Armatimonadetes, Deinococcus–Thermus, and Bacteroidetes had high correlations with other members (Figure 10B). Fungal genus *Ciliophora* (OTU2 and OTU659) and bacterial genera *Chloracidobacterium* (OTU8) and *Meiothermus* (OTU2504) played important interactive roles in their communities.

### 3.5. Comparison of Culture-Dependent and Culture-Independent Microbial Diversity Analyses

**Figure 11:**
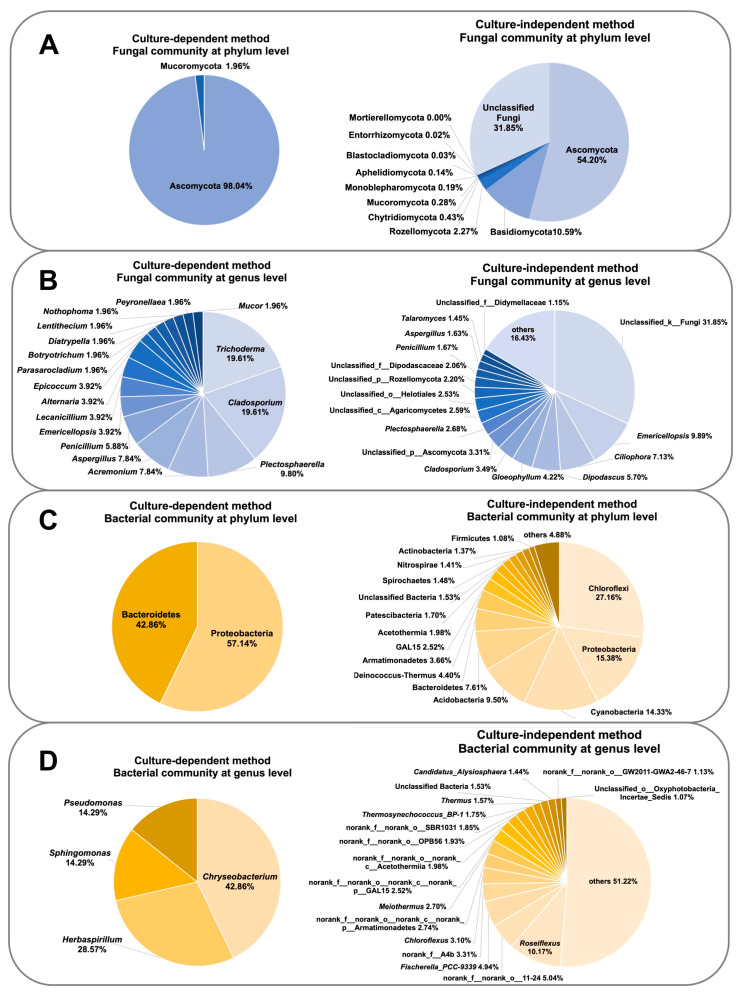
Comparison of culture-dependent and culture-independent analysis methods of the fungal community at the phylum (**A**) and genus (**B**) levels, and the bacterial community at the phylum (**C**) and genus (**D**) levels. In the taxa names, “d” = domain, “k” = kingdom, “p” = phylum, “c” = class, “o” = order, and “f” = family.

We compared the results of culture-dependent and culture-independent microbial diversity analyses performed on the 20 collected hot spring sediment samples (Figure 11). For the fungal community, only the phylum Ascomycota and Mucoromycota were found from culture-based analysis, while the other nine phyla were also detected by metabarcoding analysis (Figure 11A). Several dominant fungal genera based on the culture-independent method were also discovered from fungal isolation, including *Emericellopsis*, *Cladosporium*, *Plectosphaerella*, *Penicillium*, and *Aspergillus* (Figure 11B). For the bacterial community, culture-independent analysis showed that the studied hot springs harbored various bacterial phyla and genera, while only two phyla (Bacteroidetes and Proteobacteria) and four genera (*Chryseobacterium*, *Herbaspirillum*, *Pseudomonas*, and *Sphingomonas*) were recovered using culture-dependent analysis (Figure 11C). Isolated bacterial strains belonging to the genera *Chryseobacterium*, *Sphingomonas*, and *Pseudomonas* were also detected in the culture-independent approach, occupying very small proportions (0.00153%, 0.04868%, and 0.05038%, respectively) of the total diversity (Figure 11D). 

## 4. Discussion

This was the first study on both the fungal and bacterial ecologies and networks in sediment and water from the Julong hot springs along the active Tianchi Volcano. We provided new insights into the microbial diversity and interactions in hot spring environments using a combination of high-throughput sequencing and traditional culture-based methods, as well as into the effects of pH on the microorganism community colonizing the two studied hot spring systems. In particular, our comprehensive analysis of the whole bacterial and fungal community shed light on the fungal portion, which is an indispensable part of geothermal spring ecosystems, mostly neglected in previous studies [25].

Much more diverse and richer bacterial than fungal communities were detected in the analyzed sediments based on Illumina sequencing, thus confirming previous studies that showed bacteria to occupy the largest proportion in hot spring microbiomes [49]. Bacterial taxa belonging to the dominant identified phyla Chlorofexi, Proteobacteria, Firmicutes, Actinobacteria, Bacteroidetes, and Deinococcus–Thermus, from sediments, are typical members of microbial communities hosted by hot spring environments [49]. The dominance of the bacterial phylum Chloroflexi in the sulfur-enriched Julong hot spring sediments is consistent with the results of previous studies conducted in sulfur hot spring sediments from Odisha in East India (Atri Hot Spring), which showed similar pH values ranging from 7.42 to 8.93 [50,51]. The phylum Chloroflexi was also found dominant in the microbial mats of the Araro hot springs, located along the trans-Mexican volcanic belt [52], in co-occurrence with two other dominant phyla, Cyanobacteria and Proteobacteria, which perfectly matches our results from the Julong hot spring sediments. Among these three phyla, Chloroflexi and Cyanobacteria contain putative phototrophs commonly observed in alkaline hot springs [53], where photosynthetic bacteria are key primary producers. The dominance of the Chloroflexi and Cyanobacteria phyla in the studied hot springs confirmed their important role in supporting the network of trophic interactions between microorganisms colonizing this peculiar ecosystem, probably due to their significant contribution to carbon fixation. 

Thermophilic, alkalophilic, antimicrobial-active, and sulfide- and metal-oxidizing microbial taxonomic groups were detected, representing a clear link to the extreme properties of the studied environment, such as high temperature, alkalinity, and enrichment of metal and sulfur. Among the identified fungal genera, *Emericellopsis* occupied the largest proportion in sediments analyzed by metabarcoding. Two strains (A-S8-3 and A-S8-4) belonging to this genus were successfully isolated from the collected sediments, showing high similarity with *Emericellopsis minima* (accession number: KT290876) previously found in Bohai sea sediments, which represented the first isolation of this species in China [54]. *Emericellopsis* species have been regarded as marine-adapted fungi and extensively recorded from various marine and lake environments worldwide, including sediments from the Porcupine Bank area off the Irish coast [55], dredged sediments from the ports of Leghorn (Tuscany, Italy) [56], bottom soils of the White Sea [57], as well as sea foam and surface of freely floating decaying twigs from Windebyer Noor, a brackish lake connected to Baltic Sea in Germany [58]. *Emericellopsis* has been found to possess antimicrobial activity against plant and human pathogens, and anticancer activity [59,60]. More specifically, *Emericellopsis minima* isolated from the Talcahuano Bay of Chile was found to produce a unique fungal peptaibol (Emerimicin IV) that exhibits antibacterial activity [61]. A new bicyclic sesquiterpene was extracted from *Emericellopsis minima* isolated from marine sponge collected from the coral reef of the Similan Islands of southern Thailand [62]. Elíades et al. isolated *Emericellopsis minima* from strongly alkaline soil (pH 11) in Argentina and found this species to be alkalophilic and alkali-tolerant [63]. The dominance of *Emericellopsis* within the Julong hot springs fungal community confirmed the ability of this genus to thrive in high-selective extreme environments. The two isolated *Emericellopsis* strains from our study deserve to be further studied as potential alkalophilic fungi that may produce secondary metabolites for antimicrobial application. To the best of our knowledge, our result represents the first isolation of *Emericellopsis minima* from a hot spring habitat and provides the first record of this species in a terrestrial/fresh water ecosystem in China after the study performed by Bian et al. in the sea environment [54]. The discovery of *Emericellopsis minima* in the Julong hot springs expanded our knowledge of the ecology and distribution of this species, supporting the hypothesis of a high potential of this extremophile fungus as a source of novel compounds and bioactive metabolites. Thermophilic and sulfide- and metal-oxidizing microbial *taxa* were discovered in our study. For instance, *Roseiflexus* (Chloroflexi), the most dominant bacterial genus in the studied hot springs, is generally regarded as a filamentous anoxygenic phototrophic taxon inhabiting alkaline hot spring microbial mats [64]. Similarly, all the other abundant bacterial genera (*Fischerella*, *Chloroflexus*, *Meiothermus*, *Thermosynechococcus*, and *Thermus*) detected in our work have been frequently found associated with hot springs and described as thermophilic [65,66]. The enrichment of sulfur in the Julong hot springs may be responsible for sustaining the presence of detected photosynthetic bacteria, which are considered sulfide consumers. More specifically, some filamentous anoxygenic phototrophs such as *Chloroflexus* spp. are known to play a crucial role in the natural sulfur cycle by oxidizing sulfide to elemental sulfur [66]. The fungal genus *Plectosphaerella*, which was one of the most abundant fungal genera in the culture-dependent analysis from sediments, occupied a large proportion of the fungal community detected from high-throughput sequencing as well. All five *Plectosphaerella* isolated strains were identified as *P. cucumerina*. This fungal species is known as Mn-oxidizing and, together with the dominant bacteria *Pseudomonas* spp. identified from water samples, was previously isolated from Mn oxide deposits of the Sambe hot springs in Japan [67,68]. Mn sediments in natural environments are closely related to microbial Mn-oxidizing activity [69]. The Julong hot springs are known for their metal-rich (including Mn) spring water, and metal oxide sediments are widely distributed in the hot spring region. The *Plectosphaerella* and *Pseudomonas* strains identified in our study may possess Mn-oxidizing abilities that could be potentially applied for bioremediation of toxic metals. Further studies are needed to test this hypothesis.

Heatmaps and comparisons of the top 20 dominant microbial genera showed differential microbial patterns in the sediments of the two studied pond systems, where the abundance of a considerable number of fungal and bacterial groups were significantly different. Consistently, principal coordinate analysis revealed clear variations of fungal and bacterial communities in the two ponds of the Julong hot springs. Various environmental factors may be responsible for the community variations. To a certain extent, differences in some genera may be explained by pH. For instance, the relative abundance of the fungal genus *Emericellopsis* from metabarcoding analysis was significantly higher in Pond A, which exhibited higher pH (from 7.42 to 10.04) than Pond B (from 7.50 to 8.20). Furthermore, the two *Emericellopsis* strains from the whole study were both isolated from a single sample from Pond A (sample A-S8), which showed a noteworthy peak of pH (10.04) compared to all other samples. Although the overall fungal community, which was identified from samples whose pH mostly ranged between 7.42 and 8.68, did not show any clear correlation between pH and OTU richness, the striking community variation registered in the sole sample (A-S8) showing a pH higher than 10 speaks in favor of a strong influence of pH on the analyzed microbial diversity, at least when the environmental conditions become clearly alkaline. In fact, the sample A-S8 contributed tremendously to the total diversity of the genus *Emericellopsis*, which was overall the most dominant fungal genus in the whole analyzed hot spring fungal community. This sample yielded the highest portion of *Emericellopsis* OTUs, as well as the totality of isolates, while showing a strong reduction in the diversity and relative abundance of other fungi retrieved in our study. We may hypothesize that, in the analyzed environment, a large variety of fungi are adapted to tolerate moderately alkaline conditions, with a critical point to be set around pH 10. When the pH dramatically increases above 10, the environment becomes selective for many microorganisms, while the alkali-tolerant species dominate the microbial community. Metal concentration could be another important factor affecting the analyzed microbial community structure. The Julong hot springs are enriched with heavy metals, such as Fe and Mn. The fungal genus *Plectosphaerella* and the bacterial genus *Thermosynechococcus* have been reported to possess Mn-oxidizing [69] and iron-adapting abilities [70], respectively. The significant difference in these two genera in Pond A and Pond B may be caused by discrepant metal concentrations. Detailed chemical analyses would be necessary to disentangle the effect of the numerous elements characterizing the Julong hot spring environment on the studied microbial communities. Another possible factor to explain the different relative abundance of the photosynthetic bacterial genus *Thermosynechococcus* in the two pond systems could be the solar radiation. Nishida et al. suggested that light influences the energy conversion of phototrophs and further affects their abundance [66]. The two sampling ponds showed different openness and orientations to sunlight. Pond A was located in a completely open, flat area without shadows, while pond B was located on a more sloping area with big stones and nearly below a bridge, which may limit the sunlight availability. We may hypothesize that the different geographical locations of the two studied ponds affects the solar radiation and therefore indirectly influences the relative abundance of *Thermosynechococcus.* Further analyses of light availability in the study sites would be required to test this hypothesis. *Thermus* was also significantly more abundant in Pond B, together with *Thermosynechococcus*. The members of *Thermus* are thought to benefit from organic carbon in the form of photosynthates made available by *Thermosynechococcus* [66], thus suggesting that the presence of *Thermosynechococcus* in Pond B promotes the co-occurrence of *Thermus* and explains a significant portion of the microbial community variation in the two investigated ponds.

From fungal and bacterial network analysis, we found that significant positive interactions were dominant, thus suggesting that mutualistic relationships might be common among microorganisms coexisting in the Julong hot springs. This enables the microbes from the studied habitat to form a stable community structure against the harsh environmental conditions. However, the bacterial network showed a much more complicated structure than the fungal network. While fungi are entirely and strictly heterotrophic, some bacteria, including both phototrophic and chemotrophic species, can be autotrophic, thus acting as primary producers [66]. The contribution of autotrophic taxa and the flexibility of bacteria to explore different nutrient sources may explain the complicated mutualistic bacterial network described in this study. The phylum Chloroflexi had the highest correlation with other members in the whole bacterial network, which is consistent with the result reported by Narsing Rao et al. [18] in a study on physicochemical and microbial diversity in hot springs from three Indian provinces. Although Proteobacteria had a higher relative abundance, this phylum showed a lower level of network correlations compared to less abundant phyla, both in our study and in the previous work performed in India [18], thus suggesting a certain degree of specificity in the interactions between microbes, irrespective of their abundance. Although archaea were not the specific target of our study, it was surprising that the amplification of the 16S rRNA region with universal primers did not yield any archaeal sequences. A possible explanation could be found in the high pH of the analyzed environment. In fact, previous studies have shown that archaea are less abundant in alkaline than acidic hot springs [7,26,71]. Our results are also in accordance with a study recently conducted in Costa Rica where no archaea were detected in any hot spring at pH > 6 [72]. However, further studies are needed to understand the diversity and distribution of archaeal microorganisms in alkaline hot springs, using large scale sampling and metabarcoding analysis combined with culture-based methods, possibly by developing new culturing strategies that may allow the isolation of novel archaeal lineages. Although archaea represent a small proportion of microbes inhabiting alkaline hydrothermal systems, rarely exceeding 10% of the total microbial diversity [73], they are expected to play a pivotal role in this extreme environment for the anaerobic cycling of carbon using metabolic pathways based on fermentation, which are different from those found in bacteria [74]. Moreover, the importance of archaea in alkaline hot spring ecosystem stability could be critical at temperatures above the upper limit for photosynthesis, which is established at around 73–75 °C [75], given their capability to perform chemolithotrophic carbon fixation [76].

## 5. Conclusions

Culture-based, metabarcoding, and network analyses provided a clear picture of the fungal and bacterial diversity, community structure, and interactions in the unexplored extreme environment of the Julong geothermal springs along the active Tianchi Volcano. Typical photosynthetic, thermophilic, alkalophilic, antimicrobial-active, and sulfide- and metal-oxidizing taxonomic groups were found to characterize the unique and peculiar analyzed microbial community. The significant variation in microbial diversity observed in the two studied hot spring systems may be partly explained by the influence of pH, although further studies would be needed to disentangle the effect of different environmental factors affecting the microorganism community structure in the studied habitat. Our results indicated that mutualistic interactions may play an essential role in shaping stable microbial networks in the studied hot springs. The much more complicated bacterial than fungal network described in our study may suggest that the more flexible trophic strategies of bacteria are beneficial for their survival and fitness under extreme conditions. The various extremophile microbial strains isolated from our study may represent a precious source for the isolation of new bioactive compounds, which certainly deserve further studies to be explored.

## Figures and Tables

**Table 1:** Taxonomic groups of fungi and bacteria isolated from sediments of the Julong hot springs.

Community	Phylum	Class	Order	Genus	Number of Species	Number of Strains
**Fungi**	**Ascomycota**	Sordariomycetes	Hypocreales	*Acremonium*	2	4
				*Emericellopsis*	1	2
				*Lecanicillium*	1	2
				*Parasarocladium*	1	1
				*Trichoderma*	7	10
			Glomerellales	*Plectosphaerella*	1	5
			Sordariales	*Botryotrichum*	1	1
			Xylariales	*Diatrypella*	1	1
		Dothideomycetes	Pleosporales	*Alternaria*	2	2
				*Epicoccum*	1	2
				*Lentithecium*	1	1
				*Nothophoma*	1	1
				*Peyronellaea*	1	1
			Capnodiales	*Cladosporium*	4	10
		Eurotiomycetes	Eurotiales	*Aspergillus*	3	4
				*Penicillium*	2	3
	**Mucoromycota**	Mucoromycetes	Mucorales	*Mucor*	1	1
**Bacteria**	**Bacteroidetes**	Flavobacteriia	Flavobacteriales	*Chryseobacterium*	2	3
	**Proteobacteria**	Alphaproteobacteria	Sphingomonadales	*Sphingomonas*	1	1
		Betaproteobacteria	Burkholderiales	*Herbaspirillum*	2	2
		Gammaproteobacteria	Pseudomonadales	*Pseudomonas*	1	1

**Table 2:** Taxonomic groups of fungi and bacteria isolated from water of the Julong hot springs.

Community	Phylum	Class	Order	Genus	Number of Species	Number of Strains
**Fungi**	**Ascomycota**	Dothideomycetes	Pleosporales	*Alternaria*	3	3
			Dothideales	*Aureobasidium*	1	1
			Botryosphaeriales	*Neofusicoccum*	1	1
		Eurotiomycetes	Eurotiales	*Aspergillus*	2	6
	**Basidiomycota**	Tremellomycetes	Tremellales	*Cryptococcus*	1	2
		Microbotryomycetes	Sporidiobolales	*Sporobolomyces*	1	1
**Bacteria**	**Firmicutes**	Bacilli	Bacillales	*Bacillus*	1	1
	**Proteobacteria**	Gammaproteobacteria	Enterobacterales	*Enterobacter*	1	1
			Pseudomonadales	*Acinetobacter*	1	1
				*Pseudomonas*	8	11

## Data Availability

The fungal and bacterial DNA sequences amplified during this study are available at GenBank under accessions MZ506672–MZ506735 (fungi) and MZ497295–MZ497315 (bacteria), and in the Sequences Read Archive of NCBI as BioProject ID PRJNA744339.

## Supplementary Materials

The following are available online at https://www.mdpi.com/article/10.3390/biology10090894/s1, Figure S1: Rarefaction curves of fungal and bacterial OTUs from all samples (A,C), and from two ponds (B,D). Table S1: Fungal and bacterial diversity molecularly detected in sediments and water of Julong hot springs, from DNA extracted from isolated microbes. Table S2: An overview of fungal strains isolated from Julong hot spring sediments. Table S3: An overview of bacterial strains isolated from Julong hot spring sediments. Table S4: An overview of fungal strains isolated from Julong hot spring water. Table S5: An overview of bacterial strains isolated from Julong hot spring water. Table S6: OTU richness and Shannon index of sediment fungal and bacterial communities from the two analyzed ponds of Julong hot springs. Table S7: Network topological properties of fungal and bacterial communities in two ponds of Julong hot springs.
