# Supplementary material for: Diversity and Co-Occurrence Patterns of Fungal and Bacterial Communities from Alkaline Sediments and Water of Julong High-Altitude Hot Springs at Tianchi Volcano, Northeast China
Source: Biology (Basel). 2021 Sep 10;10(9):894. doi: 10.3390/biology10090894 (PMC8464750; doi:10.3390/biology10090894)
Supplement: Supplementary file 1 [file biology-10-00894-s001.zip › biology-1313325-supplementary.pdf]

Supplementary information

Diversity and Co-occurrence Patterns of Fungal and Bacterial Communities from Alkaline Sediments and Water of Julong High-altitude Hot Springs in Tianchi Volcano, Northeast China

Xiao Wang[1] and Lorenzo Pecoraro[1]*

[1]School of Pharmaceutical Science and Technology, Tianjin University, 92 Weijin Road, Nankai District, 300072 Tianjin, China; wang_xiao1996@163.com (X.W.); lorenzo.pecoraro@tju.edu.cn (L.P.).

*Correspondence: Lorenzo Pecoraro (telephone: +86 18520824550, e-mail: lorenzo.pecoraro@tju.edu.cn) School of Pharmaceutical Science and Technology, Tianjin University, 92 Weijin Road, Nankai District, Tianjin 300072, China.

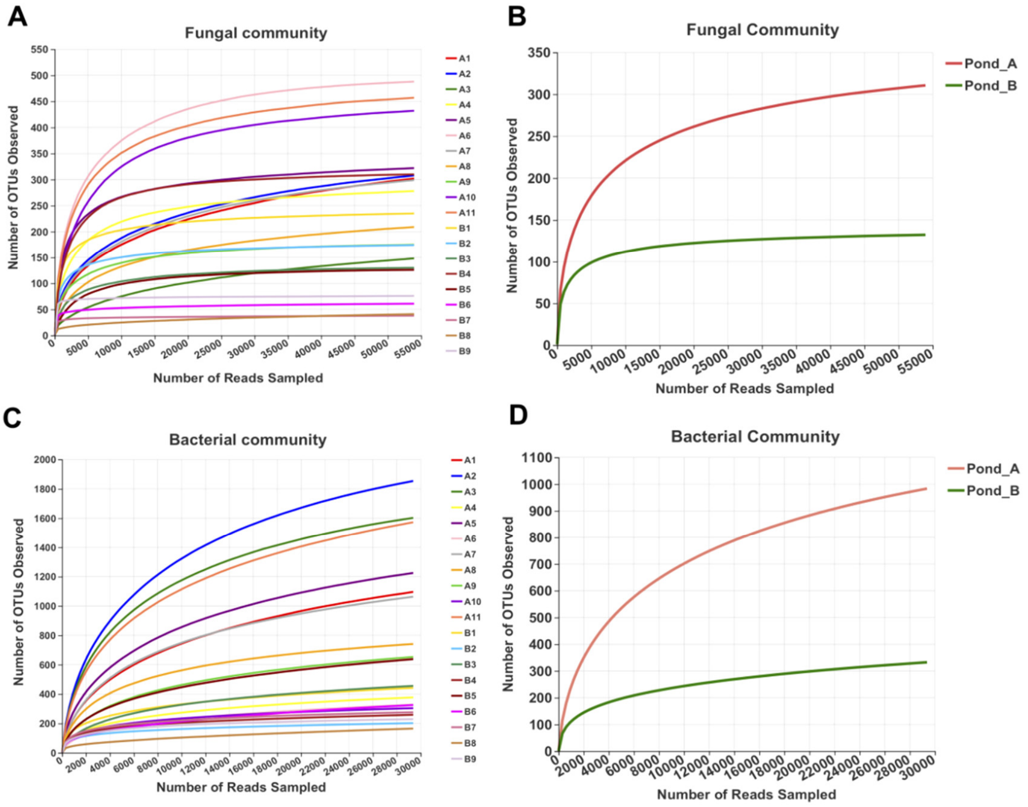

Figure S1. Rarefaction curves of fungal and bacterial OTUs from all samples (A,C), and from two ponds (B,D).

Table S1. Fungal and bacterial diversity molecularly detected in sediments and water of Julong Hot Springs, from DNA extracted from isolated microbes.

Strain	GenBank code	Best BLAST matches	Accession code	Overlap length	% match
**Fungi**					
A-S1-1	MZ506672	*Plectosphaerella cucumerina*	MW081281	817	99.12%
A-S1-2		*Peyronellaea prosopidis*	KJ159599	311	83.83%
A-S1-3	MZ506673	*Epicoccum nigrum*	MW582419	902	99.80%
A-S1-4	MZ506674	*Epicoccum nigrum*	KX099630	915	100.00%
A-S1-5	MZ506675	*Cladosporium ramotenellum*	MT529231	761	99.52%
A-S1-6	MZ506676	*Cladosporium cladosporioides*	MW582316	898	99.80%
A-S2-1	MZ506677	*Trichoderma* sp.	MK870648	998	98.42%
A-S2-2	MZ506678	Hypocreales sp.	KY612325	1032	99.71
		Acremonium sp.	KY827331	992	97.42
A-S2-3	MZ506679	Hypocreales sp.	KY612325	974	98.55%
		Acremonium sp.	KY827331	933	97.79
A-S2-4	MZ506680	*Nothophoma* sp.	MN737833	887	99.39%
A-S2-5	MZ506681	*Botryotrichum murorum*	MK990630	931	98.85%
A-S2-6	MZ506682	*Lentithecium* aff. *carbonneanum*	MW619935	924	100%
A-S2-7	MZ506683	*Trichoderma harzianum*	MK109806	1185	98.49%
A-S2-8	MZ506684	*Trichoderma harzianum*	MN944485	1057	99.48%
A-S2-9	MZ506685	Hypocreales sp.	KM066546	976	98.73%
		Acremonium sp.	KP269047	931	98.13%
A-S2-10	MZ506686	Hypocreales sp.	KM268693	944	98.51%
		Parasarocladium radiatum	MT487853	917	97.57%
A-S2-11	MZ506687	Hypocreales sp.	KY612325	959	98.53%
		Acremonium sp.	KP269047	928	98.12%
A-S3-1	MZ506688	*Penicillium oxalicum*	KT959277	981	100.00%
A-S3-2	MZ506689	*Plectosphaerella cucumerina*	MK773577	920	99.60%
A-S3-3	MZ506690	*Plectosphaerella cucumerina*	KU640393	948	100.00%
A-S3-4	MZ506691	*Plectosphaerella cucumerina*	KY781383	601	95.05%
A-S3-5	MZ506692	*Cladosporium* sp.	MK111580	881	98.99%
A-S3-6	MZ506693	*Cladosporium* sp.	KC871037	896	98.25%
A-S3-7	MZ506694	*Plectosphaerella cucumerina*	MW850542	933	100%
A-S3-8	MZ506695	*Aspergillus flavus*	KR611594	841	95.46%
A-S4-1	MZ506696	Uncultured Ascomycota	KJ461401	928	98.85%
		Aspergillus sydowii	KF706662	926	98.85%
A-S5-1	MZ506697	*Alternaria porri*	MK632003	933	99.23%
A-S7-1	MZ506698	*Mucor* sp.	KX034379	1028	97.17%
A-S7-2	MZ506699	*Trichoderma koningiopsis*	MT131273	966	98.89%
A-S7-3	MZ506700	*Trichoderma* sp.	MK871236	750	97.73%
A-S7-4	MZ506701	*Penicillium oxalicum*	MT647130	769	100.00%
A-S8-1	MZ506702	*Lecanicillium saksenae*	MW826131	946	99.05%
A-S8-2	MZ506703	*Lecanicillium saksenae*	MW826131	946	99.05%

Strain	GenBank code	Best BLAST matches	Accession code	Overlap length	% match
A-S8-3	MZ506704	Uncultured fungus	MT236953	957	98.53%
		Emericellopsis minima	KT290876	950	98.70%
A-S8-4	MZ506705	Hypocreales sp.	MT530259	1791	98.88%
		Emericellopsis minima	KT290876	1780	98.88%
A-S10-1	MZ506706	*Cladosporium cladosporioides*	MK111524	898	100.00%
A-S11-1	MZ506707	*Trichoderma harzianum*	MT065754	1029	100.00%
A-S11-2	MZ506708	*Alternaria alternata*	MH282517	955	99.81%
A-S11-3	MZ506709	*Aspergillus* sp.	MN905834	1009	100.00%
A-S11-4	MZ506710	*Trichoderma citrinoviride*	MK439516	1037	99.82%
A-S11-5	MZ506711	*Trichoderma paraviridescens*	KY750537	845	100.00%
A-S11-6	MZ506712	*Trichoderma atroviride*	HM047766	1042	99.65%
A-S11-7	MZ506713	*Trichoderma koningii*	HM037934	1026	99.82%
A-S11-8	MZ506714	*Aspergillus flavus*	MH186018	891	97.51%
B-S3-1	MZ506715	*Cladosporium cladosporioides*	MK111524	894	100.00%
B-S4-1	MZ506716	*Cladosporium cladosporioides*	HM037955	861	99.37%
B-S5-1	MZ506717	*Diatrypella pulvinata*	LC163518	832	99.56%
B-S7-1	MZ506718	*Cladosporium* sp.	MH884134	924	99.80%
B-S7-2	MZ506719	*Penicillium fimorum*	MT558942	920	99.60%
B-S8-1	MZ506720	*Cladosporium cladosporioides*	KX960912	917	99.80%
B-S8-2	MZ506721	*Cladosporium* sp.	MG835919	893	99.79%
A-W1-1	MZ506722	*Aspergillus flavus*	MH469496	870	96.92%
A-W1-2	MZ506723	Filobasidiales sp.	MK050347	1002	98.59%
		Cryptococcus sp.	HQ631032	1002	98.59%
A-W2-1	MZ506724	*Aspergillus flavus*	MH186018	885	97.32%
A-W4-1	MZ506725	Filobasidiales sp.	MK050347	1000	98.42%
		Cryptococcus sp.	HQ631032	1000	98.42%
A-W5-1	MZ506726	*Alternaria* sp.	HG530666	959	100.00%
A-W5-2	MZ506727	*Aspergillus flavus*	MT541875	662	88.43%
A-W6-1	MZ506728	*Alternaria* sp.	KX078480	939	100.00%
A-W6-2	MZ506729	*Alternaria tenuissima*	MW723828.	2172	95.28%
A-W7-1	MZ506730	*Aspergillus sydowii*	KF706662	872	96.92%
A-W7-2	MZ506731	*Aureobasidium pullulans*	JX188100	691	90.00%
A-W7-3	MZ506732	*Aspergillus sydowii*	KF706662	841	95.63%
B-W1-1	MZ506733	Uncultured fungus	KX514819	989	99.63%
		Sporobolomyces beijingensis	NR_137663	989	99.63%
B-W1-2	MZ506734	*Aspergillus flavus*	KR611594	915	97.92%
B-W3-1	MZ506735	*Neofusicoccum ribis*	EU520055	924	99.80%
**Bacteria**					
A-S5-2	MZ497295	*Chryseobacterium ureilyticum*	MN595033	774	99.30%
A-S9-1	MZ497296	*Chryseobacterium ureilyticum*	MN595033	732	99.75%
A-S9-2	MZ497297	*Chryseobacterium* sp.	MT258976	771	99.07%
A-S10-2	MZ497298	*Pseudomonas* sp.	EU482914	743	99.51%

Strain	GenBank code	Best BLAST matches	Accession code	Overlap length	% match
B-S2-1	MZ497299	Uncultured bacterium	HE586784	717	98.76%
		Sphingomonas aquatilis	KP299206	712	98.52%
B-S2-2	MZ497300	*Herbaspirillum* sp.	MT527557	761	99.29%
B-S2-3	MZ497301	Uncultured bacterium	LN524919	782	99.53%
		Herbaspirillum huttiense	KC122699	776	99.30%
A-W2-2	MZ497302	*Pseudomonas psychrophila*	MF565388	763	99.29%
A-W2-3	MZ497303	*Pseudomonas* sp.	EU482914	769	99.53%
A-W2-4	MZ497304	*Pseudomonas* sp.	EU482914	754	99.28%
A-W2-5	MZ497305	*Pseudomonas fluorescens*	MT624739	763	99.29%
A-W2-6	MZ497306	Uncultured *Pseudomonas* sp.	KJ767763	771	99.76%
A-W2-7	MZ497307	Uncultured bacterium	KM137765	780	99.08%
		Pseudomonas fluorescens	MT624739	773	98.85%
A-W4-2	MZ497308	uncultured bacterium	LR595159	776	99.53%
		Pseudomonas boreopolis	KY194239	771	99.53%
A-W5-3	MZ497309	*Pseudomonas helleri*	MK404731	769	99.53%
A-W5-4	MZ497310	*Pseudomonas* sp.	EU482914	787	99.54%
A-W5-5	MZ497311	*Pseudomonas tolaasii*	MT561438	771	98.85%
A-W7-4	MZ497312	*Pseudomonas* sp.	KY438733	765	99.52%
A-W7-5	MZ497313	*uncultured bacterium*	LR654355	760	99.06%
		Acinetobacter sp.	KY056228	760	99.06%
A-W7-6	MZ497314	*Enterobacter mori*	MT613379	743	99.04%
B-W1-3	MZ497315	*Bacillus paralicheniformis*	MT645610	750	97.92%

BLAST search closest matches of fungal internal transcribed spacer and bacterial V3−V4 hypervariable region DNA sequences amplified from Julong Hot Spring sediment and water isolated strains. In strain codes, first letters (A and B) indicate hot spring Ponds, second letters (S and W) indicate sample type (sediment and water), the following Arabic number represents the sample, while the last indicate the isolated strains. Strain GenBank accession codes, accession codes for the closest GenBank matches, sequence identity, and overlap of each match are reported.

Table S2. An overview of fungal strains isolated from Julong hot spring sediments.

Proposed Identity	Class	Order	Pond A (44)	Pond B (7)	Total (51)
**Ascomycota**					
Acremonium sp. 1	Sordariomycetes	Hypocreales	2	0	2
Acremonium sp. 2	Sordariomycetes	Hypocreales	2	0	2
Alternaria alternata	Dothideomycetes	Pleosporales	1	0	1
Alternaria porri	Dothideomycetes	Pleosporales	1	0	1
Aspergillus flavus	Eurotiomycetes	Eurotiales	2	0	2
Aspergillus sp.	Eurotiomycetes	Eurotiales	1	0	1
Aspergillus sydowii	Eurotiomycetes	Eurotiales	1	0	1
Botryotrichum murorum	Sordariomycetes	Sordariales	1	0	1
Cladosporium cladosporioides	Dothideomycetes	Capnodiales	2	3	5
Cladosporium ramotenellum	Dothideomycetes	Capnodiales	1	0	1
Cladosporium sp.1	Dothideomycetes	Capnodiales	2	0	2
Cladosporium sp.2	Dothideomycetes	Capnodiales	0	2	2
Diatrypella pulvinata	Sordariomycetes	Xylariales	0	1	1
Emericellopsis minima	Sordariomycetes	Hypocreales	2	0	2
Epicoccum nigrum	Dothideomycetes	Pleosporales	2	0	2
Lecanicillium saksenae	Sordariomycetes	Hypocreales	2	0	2
Lentithecium aff. *carbonneanum*	Dothideomycetes	Pleosporales	1	0	1
Nothophoma sp.	Dothideomycetes	Pleosporales	1	0	1
Parasarocladium radiatum	Sordariomycetes	Hypocreales	1	0	1
Penicillium fimorum	Eurotiomycetes	Eurotiales	0	1	1
Penicillium oxalicum	Eurotiomycetes	Eurotiales	2	0	2
Peyronellaea sp.	Dothideomycetes	Pleosporales	1	0	1
Plectosphaerella cucumerina	Sordariomycetes	Glomerellales	5	0	5
Trichoderma atroviride	Sordariomycetes	Hypocreales	1	0	1
Trichoderma citrinoviride	Sordariomycetes	Hypocreales	1	0	1
Trichoderma harzianum	Sordariomycetes	Hypocreales	3	0	3
Trichoderma koningii	Sordariomycetes	Hypocreales	1	0	1
Trichoderma koningiopsis	Sordariomycetes	Hypocreales	1	0	1
Trichoderma paraviridescens	Sordariomycetes	Hypocreales	1	0	1
Trichoderma sp.	Sordariomycetes	Hypocreales	2	0	2
**Mucoromycota**					
Mucor sp.	Mucoromycetes	Mucorales	1	0	1

Table S3. An overview of bacterial strains isolated from Julong hot spring sediments.

Proposed Identity	Phylum	Class	Order	Pond A (4)	Pond B (3)	Total (7)
Chryseobacterium ureilyticum	Bacteroidetes	Flavobacteriia	Flavobacteriales	2	0	2
Chryseobacterium sp.	Bacteroidetes	Flavobacteriia	Flavobacteriales	1	0	1
Herbaspirillum sp.	Proteobacteria	Betaproteobacteria	Burkholderiales	0	1	1
Herbaspirillum huttiense	Proteobacteria	Betaproteobacteria	Burkholderiales	0	1	1
Pseudomonas sp.	Proteobacteria	Gammaproteobacteria	Pseudomonadales	1	0	1
Sphingomonas aquatilis	Proteobacteria	Alphaproteobacteria	Sphingomonadales	0	1	1

Table S4. An overview of fungal strains isolated from Julong hot spring water.

Proposed Identity	Class	Order	Pond A (11)	Pond B (3)	Total (14)
**Ascomycota**					
Alternaria tenuissima	Dothideomycetes	Pleosporales	1	0	1
Alternaria sp. 1	Dothideomycetes	Pleosporales	1	0	1
Alternaria sp. 2	Dothideomycetes	Pleosporales	1	0	1
Aspergillus flavus	Eurotiomycetes	Eurotiales	3	1	4
Aspergillus sydowii	Eurotiomycetes	Eurotiales	2	0	2
Aureobasidium pullulans	Dothideomycetes	Dothideales	1	0	1
Neofusicoccum ribis	Dothideomycetes	Botryosphaeriales	0	1	1
**Basidiomycota**					
Cryptococcus sp.	Tremellomycetes	Tremellales	2	0	2
Sporobolomyces beijingensis	Microbotryomycetes	Sporidiobolales	0	1	1

Table S5. An overview of bacterial strains isolated from Julong hot spring water.

Proposed Identity	Phylum	Class	Order	Pond A (13)	Pond B (1)	Total (14)
Acinetobacter sp.	Proteobacteria	Gammaproteobacteria	Pseudomonadales	1	0	1
Bacillus paralicheniformis	Firmicutes	Bacilli	Bacillales	0	1	1
Enterobacter mori	Proteobacteria	Gammaproteobacteria	Enterobacterales	1	0	1
Pseudomonas sp. 1	Proteobacteria	Gammaproteobacteria	Pseudomonadales	3	0	3
Pseudomonas sp. 2	Proteobacteria	Gammaproteobacteria	Pseudomonadales	1	0	1
Pseudomonas sp. 3	Proteobacteria	Gammaproteobacteria	Pseudomonadales	1	0	1
Pseudomonas fluorescens	Proteobacteria	Gammaproteobacteria	Pseudomonadales	2	0	2
Pseudomonas boreopolis	Proteobacteria	Gammaproteobacteria	Pseudomonadales	1	0	1
Pseudomonas psychrophila	Proteobacteria	Gammaproteobacteria	Pseudomonadales	1	0	1
Pseudomonas helleri	Proteobacteria	Gammaproteobacteria	Pseudomonadales	1	0	1
Pseudomonas tolaasii	Proteobacteria	Gammaproteobacteria	Pseudomonadales	1	0	1

Table S6. OTU richness and Shannon index of sediment fungal and bacterial communities from the two analyzed ponds of Julong Hot Springs.

Community	α-diversity indexes	Pond A											Pond B								
		**A-S1**	**A-S2**	**A-S3**	**A-S4**	**A-S5**	**A-S6**	**A-S7**	**A-S8**	**A-S9**	**A-S10**	**A-S11**	**B-S1**	**B-S2**	**B-S3**	**B-S4**	**B-S5**	**B-S6**	**B-S7**	**B-S8**	**B-S9**
**Fungi**	OTU richness	302	308	148	278	322	488	299	209	175	432	457	235	174	130	310	126	61	38	41	76
	Shannon index	2.17	2.71	2.02	2.58	3.69	3.98	2.28	1.15	2.42	3.17	3.90	3.70	3.12	1.33	2.97	1.08	2.64	2.80	1.43	3.55
**Bacteria**	OTU richness	1096	1854	1603	377	1225	329	1063	741	653	304	1573	442	202	456	259	638	325	276	165	229
	Shannon index	4.39	6.14	5.84	3.81	5.09	2.37	4.35	4.23	3.32	3.14	5.81	4.36	3.66	2.71	3.75	3.45	3.64	3.96	2.58	3.73

Table S7. Network topological properties of fungal and bacterial communities in the two studied ponds of Julong Hot Springs.

Network metrics	Community	
	Fungi	Bacteria
Number of nodes	28	47
Total number of edges	33	299
Number and percentage of positive correlations	33 (100%)	278 (93%)
Number and percentage of negative correlations	0 (0%)	21 (7%)
Average degree	2.357	12.723
Network diameter	5	5
Average path length	2.283	2.274
